# Leukocyte telomere length and mitochondrial DNA copy number associate with endothelial function in aging-related cardiovascular disease

**DOI:** 10.3389/fcvm.2023.1157571

**Published:** 2023-06-05

**Authors:** Kangbo Li, Mengjun Dai, Mesud Sacirovic, Claudia Zemmrich, Nikolaos Pagonas, Oliver Ritter, Olaf Grisk, Lubomir T. Lubomirov, Martin A. Lauxmann, Peter Bramlage, Anja Bondke Persson, Eva Buschmann, Ivo Buschmann, Philipp Hillmeister

**Affiliations:** ^1^ Department for Angiology, Center for Internal Medicine I, Deutsches Angiologie Zentrum Brandenburg - Berlin, University Clinic Brandenburg, Brandenburg Medical School Theodor Fontane, Brandenburg an der Havel, Germany; ^2^ Charité – Universitätsmedizin Berlin, Corporate Member of Freie Universität Berlin and Humboldt-Universität zu Berlin, Berlin, Germany; ^3^ Institute for Pharmacology and Preventive Medicine, Cloppenburg, Germany; ^4^ Department for Cardiology, Center for Internal Medicine I, University Clinic Brandenburg, Brandenburg Medical School Theodor Fontane, Brandenburg an der Havel, Germany; ^5^ Faculty of Health Sciences Brandenburg, Joint Faculty of the Brandenburg University of Technology Cottbus – Senftenberg, The Brandenburg Medical School Theodor Fontane, University of Potsdam, Brandenburg an der Havel, Germany; ^6^ Institute of Physiology, Brandenburg Medical School Theodor Fontane, Neuruppin, Germany; ^7^ Institute of Biochemistry, Brandenburg Medical School Theodor Fontane, Brandenburg an der Havel, Germany; ^8^ Department of Cardiology, University Clinic Graz, Graz, Austria

**Keywords:** telomere length, mitochondrial DNA copy number, endothelial function, flow-mediated dilation, peripheral blood mononuclear cells, cell-free DNA, aging-related cardiovascular disease

## Abstract

**Background:**

We investigated the association between leukocyte telomere length, mitochondrial DNA copy number, and endothelial function in patients with aging-related cardiovascular disease (CVD).

**Methods:**

In total 430 patients with CVD and healthy persons were enrolled in the current study. Peripheral blood was drawn by routine venipuncture procedure. Plasma and peripheral blood mononuclear cells (PBMCs) were collected. Cell-free genomic DNA (cfDNA) and leukocytic genomic DNA (leuDNA) were extracted from plasma and PBMCs, respectively. Relative telomere length (TL) and mitochondrial DNA copy number (mtDNA-CN) were analyzed using quantitative polymerase chain reaction. Endothelial function was evaluated by measuring flow-mediated dilation (FMD). The correlation between TL of cfDNA (cf-TL), mtDNA-CN of cfDNA (cf-mtDNA), TL of leuDNA (leu-TL), mtDNA-CN of leuDNA (leu-mtDNA), age, and FMD were analyzed based on Spearman's rank correlation. The association between cf-TL, cf-mtDNA, leu-TL, leu-mtDNA, age, gender, and FMD were explored using multiple linear regression analysis.

**Results:**

cf-TL positively correlated with cf-mtDNA (*r* = 0.1834, *P* = 0.0273), and leu-TL positively correlated with leu-mtDNA (*r* = 0.1244, *P* = 0.0109). In addition, both leu-TL (*r* = 0.1489, *P* = 0.0022) and leu-mtDNA (*r* = 0.1929, *P* < 0.0001) positively correlated with FMD. In a multiple linear regression analysis model, both leu-TL (*β* = 0.229, *P* = 0.002) and leu-mtDNA (*β* = 0.198, *P* = 0.008) were positively associated with FMD. In contrast, age was inversely associated with FMD (*β* = −0.426, *P* < 0.0001).

**Conclusion:**

TL positively correlates mtDNA-CN in both cfDNA and leuDNA. leu-TL and leu-mtDNA can be regarded as novel biomarkers of endothelial dysfunction.

## Introduction

Current trends in life expectancy and concomitant demographic change (1) are leading to an ever-increasing number of patients suffering from aging-related disease (ARD), in which, cardiovascular disease (CVD) remains the major cause of death in the elderly population worldwide (2). The dominant cause of aging-related CVD is atherosclerosis (AS), which refers to the pathological process in that intimal lipids and fibrous elements encroach on the lumen of large arteries (3). In this context, an accumulating body of research has highlighted that cellular senescence in AS are characterized by telomere attrition (4) and mitochondrial DNA depletion (5).

Indeed, telomeres and mitochondria play critical roles in premature biological aging (6) and aging-related CVD (7). More precisely, a telomere is a specific region of repetitive nucleotide sequences associated with specialized proteins at the termini of linear chromosomes. Telomeres protect the genome from nucleolytic degradation and interchromosomal fusion, thereby ensuring the integrity of linear chromosomes (8). Telomere attrition occurs during each DNA replication and ultimately triggers the senescence and apoptosis in cells. Therefore, telomere length (TL) has been considered a biological marker of aging (9–11). In terms of CVD, although atherosclerotic lesion develops focally, it usually results in chronic systemic inflammation, which increases the turnover and biological age of vascular cells (10) and circulating cells (12). Therefore, TL can be regarded as an individual prognostic marker for cardiovascular risk prediction (13).

A mitochondrion is a double-membrane-bound organelle, which can be found in most eukaryotic organisms. Mitochondria generate the majority of adenosine triphosphate (ATP) during aerobic respiration, thereby playing a critical role in cellular energy production (14). ARD is attributed to the deleterious effects of reactive oxygen species (ROS) on various cell components (15). Since the majority of ROS are generated by the mitochondrial electron transport chain (16), mitochondrial DNA is more prone to damage by ROS (17). Thus, the free radical theory was refined as the mitochondrial theory of ageing (15). Likewise, recent studies show that mitochondrial DNA damage widely occurs in both the vascular and circulating cells (5). Therefore, mitochondrial DNA copy number (mtDNA-CN) can be also regarded as an individual prognostic marker for cardiovascular risk prediction (18).

Endothelial function reflects the production of endothelium-derived factors that regulate cardiovascular homeostasis, such as vascular tone, blood flow and blood pressure (19). In contrast, endothelial dysfunction (ED) is a systemic pathological state characterized by imbalanced vasodilation and vasoconstriction of the endothelium (20). There is no doubt that AS is the leading cause of CVD (3), yet, numerous studies have shown that ED precedes the angiographic or ultrasonic evidence of AS in aging-related CVD (21, 22). It has been shown that ED accompanies multifactorial endothelial aging (23, 24). Beyond that, both telomeres and mitochondria may be essential for the key aspects of endothelial function (25, 26). Yet, the association between TL, mtDNA-CN and endothelial function is still unclear.

In this regard, flow-mediated vasodilatation (FMD) is the most widely used non-invasive approach for assessment of endothelial function by measuring the ability of the arteries respond to endothelium-derived nitric oxide (NO) during reactive hyperemia. NO-dependent vasodilation can be quantified as an index of vasomotor (endothelial) function. Furthermore, both circulating cell-free DNA and peripheral blood mononuclear cells (PBMCs) are widely used in aging research as they have reasonable prognostic or diagnostic potential (27, 28). Cell-free DNA is the fragmented double-strand DNA released from dying cells in circulating blood (29). In contrast, PBMCs consist of lymphocytes and monocytes, which are subsets of leukocytes (30). In this study, we analyzed TL and mtDNA-CN from both cell-free and leukocytic genomic DNA, and investigated the potential relationships between TL, mtDNA-CN and endothelial function in aging-related CVD.

## Materials and methods

### Study population

The WalkByLab registry (www.walkbylab.com) is an ongoing CVD screening trial. It aims to screen, diagnose and follow up patients with CVD in the non-metropolitan areas of the federal state of Brandenburg, Germany. A structured multimodal risk factor management standard has been set for measurement and assessment of vascular function in the WalkByLab (31). More than 1,000 participants have been examined in the WalkByLab subcenter of Brandenburg (University Clinic Brandenburg) from June 2018 to December 2022. Here, blood samples of 430 participants were randomly selected and used for the current study.

### Isolation of plasma and peripheral blood mononuclear cells

Around 6 ml of peripheral blood were collected in the BD Vacutainer EDTA Blood Collection Tube (Becton Dickinson). Then, blood was transferred into a 50-ml conical centrifuge tube, an equal volume of 1 × PBS was added and mixed gently. Diluted blood was slowly layered onto the Ficoll-Paque density gradient media (GE Healthcare) at a ratio of 4:3, and centrifuged 25 min at room temperature (400 × g without brake). The upper layer of plasma was collected and immediately frozen at −80°C until genomic DNA extraction. The mononuclear cell layer was transferred into a new conical centrifuge tube and centrifuged 10 min at 450 × g. Supernatant was removed, the cell pellet was vortexed immediately after adding 1.5 ml erythrocyte lysis buffer (PAN Biotech GmbH), and then incubated in the dark at room temperature for 15 min. After washing with PBS, the cell pellet was immediately frozen at −80°C until genomic DNA extraction.

### Cell-free and leukocytic genomic DNA extraction

For cell-free genomic DNA (cfDNA) extraction, 2 ml frozen plasma was thawed and centrifuged at room temperature for 10 min (20,000 × g) to remove cell debris. cfDNA was extracted by using QIAamp DNA Blood Mini Kit (Qiagen). For leukocytic genomic DNA extraction, frozen PBMCs pellet including 1 × 10^6^ cells was thawed in 1 ml PBS at room temperature. Leukocytic genomic DNA (leuDNA) was extracted by using the DNeasy Blood & Tissue Kit (Qiagen). Quantitative analysis of genomic DNA (gDNA) was performed by using the Nanodrop™ Microvolume Spectrophotometer (Thermo Fisher Scientific). cfDNA and leuDNA were further diluted to final concentrations of 1 ng/µl and 10 ng/µl, respectively.

### Analyses for telomere length and mitochondrial DNA copy number

For quantitative polymerase chain reaction (qPCR) amplification of cfDNA template, each reaction system contained 25 µl gDNA, 2 µl primer working solution, and 25 µl PowerTrack™ SYBR Green Master Mix (Thermo Fisher Scientific). For qPCR amplification of leuDNA template, each reaction system contained 5 µl gDNA, 1 µl primer working solution, 4 µl RNase/DNase-free water and 10 µl PowerTrack™ SYBR Green Master Mix (Thermo Fisher Scientific). 60 cycles of a two-step qPCR were performed. All of primers used in this study were synthesized by the Eurofins Genomics Germany GmbH. Primer sequences are shown in Table 1.

**Table 1:** List of qPCR primers sequences.

Gene	Forward	Reverse
TELO	GGTTTTTGAGGGTGAGGGTGAGGGTGAGGGTGAGGGT	TCCCGACTATCCCTATCCCTATCCCTATCCCTATCCCTA
36B4	CAGCAAGTGGGAAGGTGTAATCC	CCCATTCTATCATCAACGGGTACAA
MITO	CACTTTCCACACAGACATCA	TGGTTAGGCTGGTGTTAGGG
B2M	TGTTCCTGCTGGGTAGCTCT	CCTCCATGATGCTGCTTACA

TELO, telomeric DNA; 36B4, acidic ribosomal phosphoprotein PO; MITO, mitochondrial DNA; B2M, Beta-2-Microglobulin.

Telomere length (TL) was expressed as telomeric DNA (teloDNA) relative to acidic ribosomal phosphoprotein PO (36B4), 36B4 is a single copy gene and serves as internal reference. Here, TL was calculated according to the formula: TL = 2^−ΔCT^, ΔCT = CT_teloDNA_−CT_36B4_. Similarly, mitochondrial copy number (mtDNA-CN) was expressed as mitochondrial DNA relative to a single copy gene β2 microglobulin (B2M), B2M is a single copy gene and serves as internal reference. Here, mtDNA-CN was calculated according to the formula: mtDNA-CN = 2 × 2^−ΔCT^, and ΔCT = CT_mtDNA_−CT_B2M_. Data were expressed as a relative level by normalizing against mean value.

### Evaluation of endothelial function by flow-mediated dilation

Endothelial function was evaluated by measuring FMD using AngioDefender™ system (Everist Health). The AngioDefender™ enables automatic and non-invasive measurement of brachial FMD. In brief, a proprietary software algorithm was used to analyze the high resolution continuous electrocardiogram-gated B-mode ultrasound imaging during reactive hyperemia in brachial artery. The equivalence of FMD determined by the AngioDefender™ and the classical ultrasound or Doppler flow based analysis has been verified (32).

### Statistical analysis

All statistical analyses were performed by using IBM SPSS26 or R language. Clinical characteristics were given as mean ± standard deviation (SD), relative TL and mtDNA-CN were given as mean ± standard error of the mean (SEM). Correlation between variables was analyzed using Spearman's correlation coefficient. Three multiple linear regression analysis models (stepwise method) were used to investigate the relationship between major variables. *P* ≤ 0.05 was considered to indicate statistical significance.

## Results

### Clinical characteristics

The mean age of 430 participants was 68.9 years. Specifically, 8 (1.9%) participants were between the age of 25 and 44 years (young age). 58 (13.5%) participants were between the age of 45 and 59 years (middle age). 226 (52.6%) participants were between the age of 60 and 74 years (elderly age). 138 (32.1%) participants were between the age of 75 and 89 years (senile age), respectively.

The mean FMD of 430 participants was 7.07 (±2.50) %. Specifically, the FMD of 111 participants were less than or equal to 5.50% (endothelial dysfunction), mean (±SD): 4.04 (±0.99) %. The FMD of 318 participants were more than 5.50% (normal endothelial function), mean (± SD): 8.13 (± 1.95) %. All other clinical characteristics are presented in Table 2.

**Table 2:** Clinical characteristics.

Characteristics	*n* (%)
Male	221 (51.4%)
Smoking	165 (38.4%)
CAD	168 (39.1%)
PAD	174 (40.5%)
CeVD	44 (10.2%)
Hypertension	307 (71.4%)
DM	85 (19.8%)
MI	61 (14.2%)
HF	136 (31.6%)
RI	67 (15.6%)
Aspirin	177 (41.2%)
P_2_Y_12_i	51 (11.9%)
Anticoagulants	85 (19.8%)
ACEis	129 (30.0%)
ARBs	138 (32.1%)
β-blockers	194 (45.1%)
CCB	120 (27.9%)
Diuretics	129 (30.0%)
Digitalis	8 (1.9%)
Statins	222 (51.6%)
Antidiabetics/Insulins	71 (16.5%)

CAD, coronary artery disease; PAD, peripheral arterial disease; CeVD. cerebrovascular disease; DM, diabetes mellitus; MI, myocardial infarction; HF, heart failure; RI, renal insufficiency; P_2_Y_12_i, P_2_Y_12_ inhibitors; ACEis, angiotensin-converting enzyme inhibitors; ARBs, angiotensin II receptor blockers; CCBs, calcium channel blockers.

### Flow-mediated dilation inversely correlates with age

**Figure 1:**
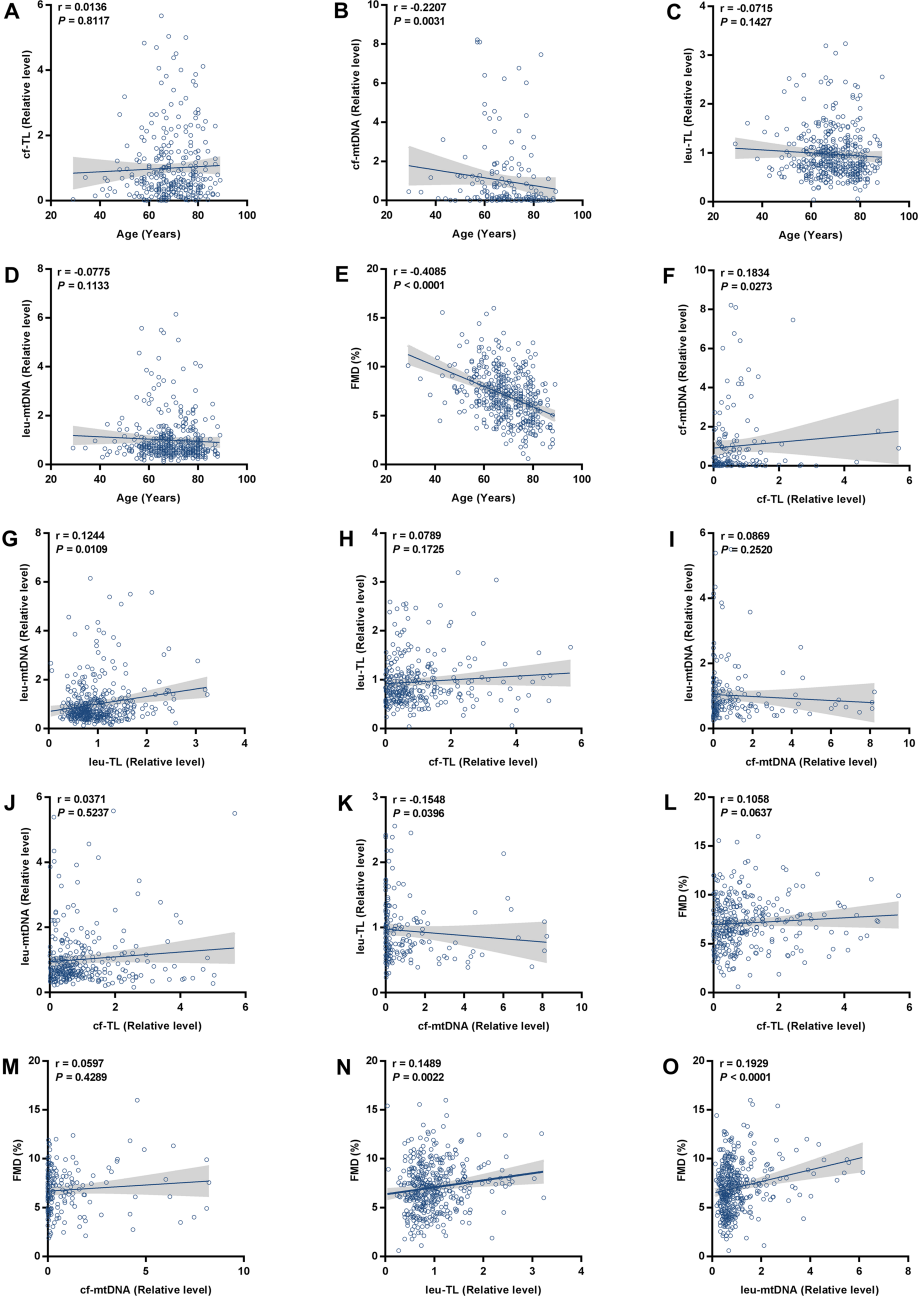
Correlation analyses. (A-E) Correlation analyses between cf-TL, cf-mtDNA, leu-TL, leu-mtDNA, FMD, and age. (F-K) Correlation analyses between cf-TL, cf-mtDNA, leu-TL, and leu-mtDNA. (L-O) Correlation analyses between cf-TL, cf-mtDNA, leu-TL, leu-mtDNA, and FMD.

First, we analyzed the correlation between cf-TL, cf-mtDNA, leu-TL, leu-mtDNA, FMD, and age. Here, a significant inverse correlation existed between cf-mtDNA and age (*r* = −0.2207, *P* = 0.0031) (Figure 1B). Besides, cf-TL slightly positively correlated with age (Figure 1A), while both leu-TL and leu-mtDNA inversely correlated with age (Figures 1C,D). However, these results were without any statistical significance. Furthermore, a significant inverse correlation existed between FMD and age (*r* = −0.4085, *P* < 0.0001) (Figure 1E).

### Telomere length positively correlates with mitochondrial DNA copy number in both cell-free and leukocytic genomic DNA

Then, we analyzed the correlation between cf-TL, cf-mtDNA, leu-TL, and leu-mtDNA. Here, cf-TL positively correlated with cf-mtDNA (*r* = 0.1834, *P* = 0.0273) (Figure 1F). Similarly, leu-TL positively correlated with leu-mtDNA (*r* = 0.1244, *P* = 0.0109) (Figure 1G). Besides, positive correlations existed between cf-TL and leu-TL, and also, between cf-mtDNA and leu-mtDNA (Figures 1H,I). However, a significant difference was not reached. In addition, there was a significant inverse correlation between cf-mtDNA and leu-TL (*r* = −0.1548, *P* = 0.00396) (Figure 1K).

### Leukocyte telomere length and mitochondrial DNA copy number positively correlate with flow-mediated dilation

Finally, we analyzed the correlation between cf-TL, cf-mtDNA, leu-TL, and leu-mtDNA, with FMD. Here, both leu-TL (*r* = 0.1489, *P* = 0.0022) and leu-mtDNA (*r* = 0.1929, *P* < 0.0001) positively correlated with FMD (Figures 1N,O). Although both cf-TL and cf-mtDNA positively correlated with FMD, no significant difference was reached (Figures 1L,M).

### Multivariate correlates of flow-mediated dilation

**Figure 2:**
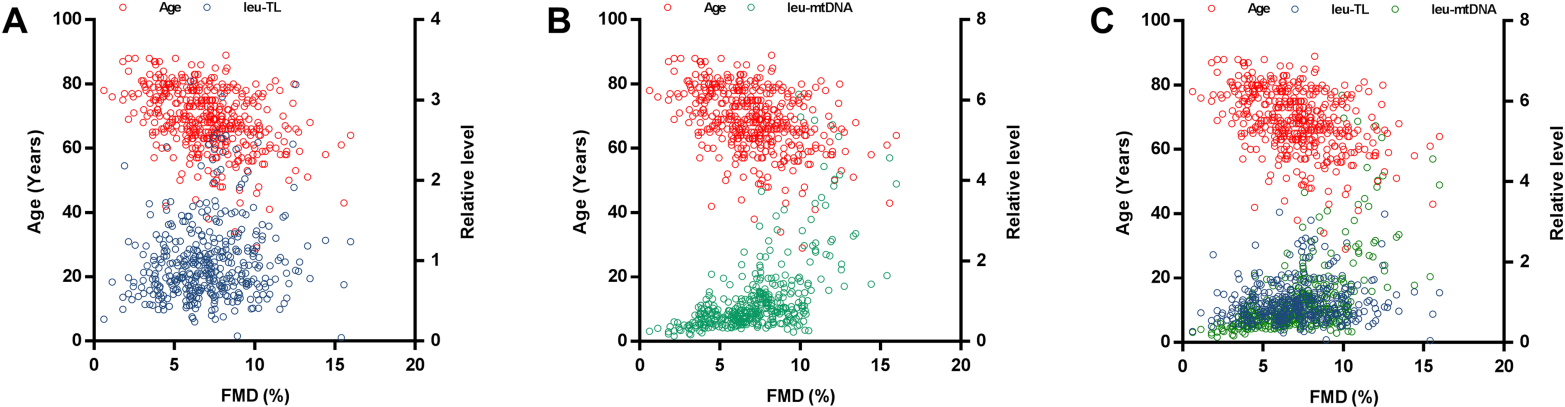
Multiple linear regression analyses. (**A**) Multiple linear regression analysis between leu-TL, age, and FMD. (**B**) Multiple linear regression analysis between leu-mtDNA, age, and FMD. (**C**) Multiple linear regression analysis between leu-TL, leu-mtDNA, age, and FMD.

Adjusted covariates in model 1 consisted of cf-TL, leu-TL, age and gender (male). Here, leu-TL was positively associated with FMD (*β* = 0.239, SE = 0.148, *P *< 0.0001), while age was inversely associated with FMD (*β* = −0.419, SE = 0.013, *P *< 0.0001). Variates of cf-TL and gender (male) were excluded in this stepwise model (Table 3 and Figure 2A).

**Table 3:** Multiple linear regression analyses between leu-TL, leu-mtDNA, and age with FMD.

Variables	Model 1			Model 2			Model 3		
	*β*	SE	*P*	*β*	SE	*P*	*β*	SE	*P*
leu-TL	0.239	0.148	<0.0001	–	–	–	0.229	0.195	0.002
leu-mtDNA	–	–	–	0.188	0.350	0.007	0.198	0.394	0.008
Age	−0.419	0.013	<0.0001	−0.369	0.015	<0.0001	−0.426	0.016	<0.0001

Model 1: variables consisted of leu-TL, cf-TL, age, and gender (male). Model 2: variables consisted of leu-mtDNA, cf-mtDNA, age, and gender (male). Model 3: variables consisted of leu-TL, cf-TL, leu-mtDNA, cf-mtDNA, age, and gender (male). SE, standard error.

Adjusted covariates in model 2 consisted of cf-mtDNA, leu-mtDNA, age and gender (male). Here, leu-mtDNA was positively associated with FMD (*β* = 0.188, SE = 0.350, *P *= 0.007), while age was inversely with FMD (*β* = −0.369, SE = 0.015, *P *< 0.0001). Variates of cf-mtDNA and gender (male) were excluded in this stepwise model (Table 3 and Figure 2B).

Adjusted covariates in model 3 consisted of cf-TL, leu-TL, cf-mtDNA, leu-mtDNA, age and gender (male). Here, both leu-TL (*β* = 0.229, SE = 0.195, *P *= 0.002) and leu-mtDNA (*β* = 0.198, SE = 0.394, *P *= 0.008) were positively associated with FMD. Again, age was inversely associated with FMD (*β* = −0.426, SE = 0.016, *P *< 0.0001). Variates of cf-TL, cf-mtDNA and gender (male) were excluded in this stepwise model (Table 3 and Figure 2C).

## Discussion

First of all, our results showed that FMD inversely correlated with age in the patients with CVD. Consistent with our results, numerous studies have shown that FMD declines with age, which is an independent determinant of endothelial function (33–36). The endothelium is one of the largest human organs by area alone, and it interacts with nearly every system in the body (37). It is well accepted that age steadily impairs endothelial function through downregulating endothelial nitric oxide synthase (NOS) expression, inhibiting of NOS activity, and increasing NO degradation (38). Although the pathophysiology of age-dependent ED has not been fully revealed, a cause-and-effect relationship between diminished NO and ED has been confirmed (39, 40).

In addition, our results also indicate that both leu-TL and leu-mtDNA inversely correlate with age, yet a significant difference was not reached. One possible explanation could be that much more elderly aged participants than young and middle aged participants were recruited in this study. In fact, age-associated telomere attrition is a generally accepted finding based on numerous studies. In this regard, Nordfjäll et al. observed an age-related blood cell TL attrition with an interval of one-decade from 959 individuals (41). Spyridopoulos et al. demonstrated that leu-TL correlated with the progress of CVD, and it can be shown in all leukocyte populations, including peripheral blood stem cells and progenitor cells (42). Furthermore, Lee et al. reported that PBMC telomere fluorescence intensity was significantly decreased with age in healthy cynomolgus monkeys (43).

With regard to mtDNA-CN, Mengel-From et al. observed a tendency of fewer PBMC mtDNA-CN with aging by analyzing 1,067 subjects from a Danish cohort study (44). Furthermore, Foote et al. reported that arterial mtDNA-CN decreased with aging in mice (45). Indeed, because mtDNA is the major target of aging-associated mutation, age independently affects mtDNA-CN (46) and mitochondrial function (47).

Beyond that, an unexpected result was that cf-mtDNA inversely correlated with age. Here, it is well known that cf-mtDNA fragments are released extracellularly when dysfunctional mitochondria are accumulated in senescent cells (48). Therefore, cf-mtDNA fragments can be regarded as an aging biomarker (49). In this regard, Pinti et al. demonstrated that cf-mtDNA and proinflammatory cytokines increased gradually with age, which suggested that cf-mtDNA acts as the damage-associated molecular pattern in this context (50, 51). Furthermore, Ampo et al. demonstrated that cf-mtDNA was significantly increased in frail elderly subjects (52). However, these results are in contrast with our finding from the current study, the mechanism is still unclear.

**Figure 3:**
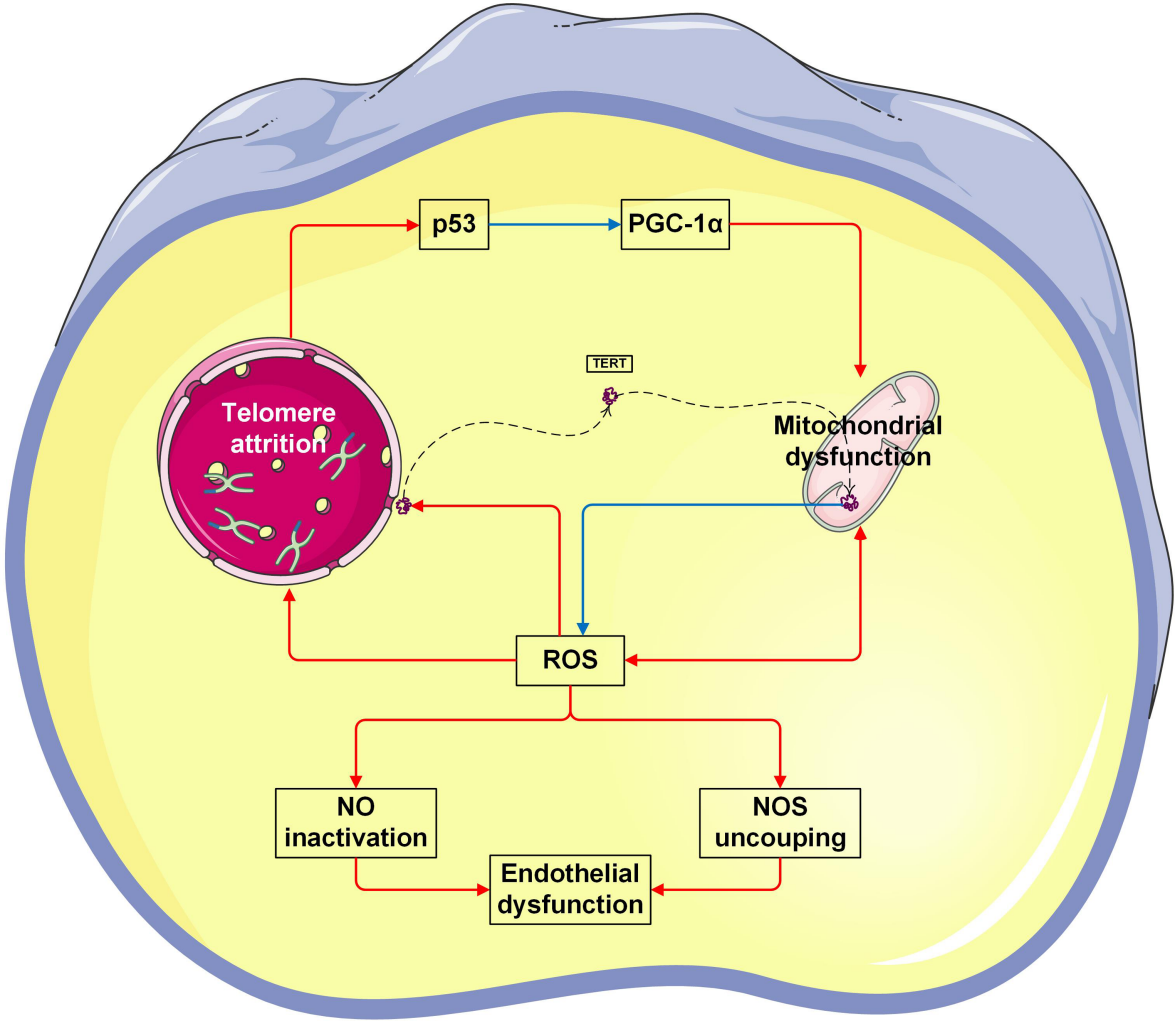
Vicious cycle between telomere attrition, mitochondrial dysfunction, and endothelial dysfunction. The p53 protein is a transcription factor responsible for preserving genomic integrity (69). Peroxisome proliferator-activated receptor gamma coactivator 1-alpha (PGC-1α) is a transcription factor responsible for regulating mitochondrial biogenesis (70). In the case of telomere attrition, p53 inhibits PGC-1α, thereby leading to mitochondrial dysfunction. Vice versa, mitochondrial-derived ROS causes telomeric DNA damage, thereby leading to telomere attrition (61, 71). Telomerase reverse transcriptase (TERT) is the catalytic subunit of telomerase. In response to ROS, TERT is exported from the nucleus. In mitochondria, TERT binds to and protects mtDNA, thereby improving electron transport chain function and reducing ROS generation (72). On the other hand, excessive ROS suppresses the production of bioactive NO levels, but increases the production of by toxic peroxynitrite. Peroxynitrite uncouples endothelial NOS to form a dysfunctional superoxide-generating enzyme (73). Taken together, both NO inactivation and NOS uncoupling are enrolled in endothelial dysfunction (74). The Figures were partly generated using Servier Medical Art, provided by Servier, licensed under a Creative Commons Attribution 3.0 unported license.

This study showed a significant positive correlation between TL and mtDNA-CN in both cell-free and leukocyte genomic DNA. To the best of our knowledge, it is the first time to characterize the intimate relationship of TL and mtDNA-CN in patients with CVD. Specifically, higher levels of leu-TL and leu-mtDNA may indicate physiological condition or a compensation stage (11), while higher levels of cf-TL and cf-mtDNA may indicate pathological condition or a decompensation stage (50, 53). Here, a mounting number of studies have been performed to provide evidence that TL and mtDNA are coordinately regulated (54–57). Furthermore, co-regulation of telomeres and mitochondria play an important role in the pathophysiology process of chronic diseases and senescence (58, 59). Indeed, the interplay between telomeres and mitochondria was confirmed in recent studies (60). Therefore, the “telomere-mitochondrial axis” was proposed (61), which may serve as a target of molecular damage in aging (62) (Figure 3).

On the one hand, mitochondrial dysfunction leads to telomere attrition (63). During aging, damaged mitochondria produce indiscriminate amounts of ROS, which is known to cause irreversible damage to DNA by oxidizing cellular constituents (64). As a consequence, the normal redox signaling is disrupted and oxidative stress occurs (65). Accordingly, ROS also damages telomeric DNA. It has been shown that telomere attrition is largely caused by the repair inefficiency of a specific telomeric DNA single-strand (66). Therefore, mitochondrial dysfunction contributes to telomere attrition (67, 68). Here, Sanderson et al. demonstrated that telomere attrition in CD8^+^ T cells was suppressed by a ROS scavenger (67). Besides, Liu et al. demonstrated that telomere attrition in murine embryos was prevented by an antioxidant (68).

The other way around, telomere attrition leads to mitochondrial dysfunction (63). Mitochondrial content is regulated by mitochondrial biogenesis and mitophagy (75). Since mitochondria are sensitive to environmental cues, mitochondrial biogenesis could also be repressed due to telomere damage (59, 71). Telomere attrition induces DNA damage (6, 76, 77), thereby decreasing mtDNA-CN, while increasing ROS (6), which in turn damages both telomeres and mitochondria (59, 71). In addition, it has been known that p53 protein is a transcription factor responsible for preserving genomic integrity (69), and peroxisome proliferator-activated receptor gamma coactivator 1-alpha (PGC-1α) is a transcription factor responsible for regulating mitochondrial biogenesis (70). In the case of telomere attrition, p53 inhibits PGC1α, thereby leading to mitochondrial dysfunction.

Furthermore, telomerase, an enzyme responsible for maintenance of telomere length by synthesizing terminal DNA, also appears to have the function of protecting against oxidative stress. In contrast, a mutant telomerase induces high levels of mitochondrial ROS, and thereby leading to mitochondrial dysfunction (78) (Figure 3).

Beyond that, this study showed that both leu-TL and leu-mtDNA, rather than cf-TL and cf-mtDNA, are positively associated with FMD in patients with aging-related CVD. Indeed, levels of leu-TL and leu-mtDNA may indicate the capacities of compensation (11). It has been demonstrated that telomeres and mitochondria (79) from immune cells play critical roles in peripheral arterial disease (80) and heart failure (81), respectively. In contrast, while cf-TL and cf-mtDNA may indicate stages of decompensation (50, 53). Therefore, both cf-TL and cf-mtDNA have been regarded as new biomarkers in cancer diagnosis and treatment (82–84). Yet, inverse linear correlations were not observed between cf-TL, cf-mtDNA and FMD in our current study.

Regarding the correlation between leu-TL and FMD, similar studies have been performed by Eguchi et al. (85) and Nakashima et al. (86), respectively. However, a significant correlation between leu-TL and FMD was not confirmed in their studies (85, 86). Nezu et al. reported that telomere G-tail length, but not total leu-TL, positively associated with FMD (87). Besides, Combrink et al. demonstrated that leu-TL positively correlated with plasma nitrite/nitrate levels in a bi-ethnic study (88), which are partially in support of our findings.

To date, former research mainly focused on telomerase activity and endothelial function. Here, it has been reported that activation of telomerase restored endothelial function in the human coronary and adipose arterioles (89). In addition, Bhayadia et al. demonstrated that endothelium-dependent vasodilation in telomerase deficient mice was impaired, which can be further restored by inhibiting oxidative stress (90).

It has been well accepted that endothelial function is NO-dependent, which fulfills a wide range of biological functions in cardiovascular homeostasis (39). NO inhibits telomere attrition by modulating telomerase activity (91). In fact, endothelial senescence and systemic vascular dysfunction are results of disruption of the delicate balance between NO and ROS (90, 92, 93). Therefore, a causal relationship between endothelial function and TL can be speculated based on our results (Figure 3).

Regarding the relationship between leu-mtDNA and FMD, Fetterman et al. reported that PBMC mitochondrial DNA damage inversely correlated to FMD in patients with diabetes mellitus and CVD, but a significant difference was not reached (94). In addition, Kakarla et al. demonstrated that mitochondrial membrane protein levels were positively associated with FMD in patients with type 2 diabetes mellitus (95).

Mitochondrial content is regulated by both mitochondrial biogenesis and mitophagy. Upregulation of mitochondrial biogenesis leads to increased mtDNA-CN correspondingly, thereby resulting in an enhanced metabolic capacity (96). In particular, endothelial mitochondrial energy production plays an important role in of vascular tone regulation (82). Research has shown that that mtDNA-CN negatively correlated with the disease severity and duration in healthy subjects (44, 97).

However, some research also indicated that mtDNA-CN positively correlated to cancer and CVD risk (98–100). Because the mtDNA is prone to DNA damage and susceptible to oxidative stress, increase of mtDNA-CN may serve as a possible compensatory mechanism to cope with mitochondrial dysfunction (101). In this regard, it has been reported that increased mtDNA-CN of gastrocnemius muscle was associated with lower ankle brachial index in patients with peripheral artery disease (100). In fact, within a certain level, ROS may increase mitochondria abundance and mtDNA content, thereby compensating for defective mitochondria to uphold the energy metabolism. Once beyond a threshold, ROS causes oxidative damage to mtDNA and elicits an irreversible apoptosis (102). Yet, the inner link between mitochondria in immune cells and endothelial function is still poorly understood. In conclusion, further studies are needed to investigate the putative causal relationship of mitochondria of immune cells and endothelial function in patients with CVD.

## Conclusions

First, both leu-TL and mtDNA-CN positively correlate with FMD, while FMD negatively correlates with age. Second, TL positively correlates with mtDNA-CN in both leukocytic and cell-free genomic DNA. In conclusion, leu-TL and leu-mtDNA can be regarded as novel biomarkers of aging-related CVD.

## Limitations

Because cfDNA is the fragmented double-strand DNA released from dying cells in circulating blood (29), the DNA concentrations were very low. Thus, the relevant Cq values in qPCR analysis were comparatively high, and finally affected the reliability of this study.

## Data Availability

The original contributions presented in the study are included in the article/**Supplementary Materials**, further inquiries can be directed to the corresponding author/s.
